# The Envelope, Please

**DOI:** 10.1100/tsw.2000.8

**Published:** 2000-10-13

## Abstract

Nobel Prize announcements dominated science news this week. The Nature news section led with three Prize-winner stories: medicine, physics, and chemistry. Science saved the Nobel details for next week and instead led with an announced $58 million non-profit effort to sequence the mouse genome and distribute the results free of charge.

*Nature* reports that, in a surprising turn of events, eight white men of European descent and one Japanese man were awarded Nobel Prizes in science this week. The award for Medicine and Physiology goes to Arvid Carlsson of the University of Gothenburg in Sweden, Paul Greengard of the Rockefeller University in New York, and Eric Kandel of Columbia University in New York, for their pioneering studies of conversing neurons. In particular, Carlsson discovered that the chemical dopamine is a brain neuro-transmitter that must stay in near-perfect balance to prevent schizophrenia (too much) and Parkinson's disease (too little). Greengard added detailed maps of multi-syllabic dopamine pathways and Kandel described how changing neuro-transmitter concentrations assisted learning.

Computers and telecommunications held sway over the physical science prizes. The Physics Prize was shared by Zhores Alferov of the Ioffe Physico-Technical Institute in St Petersburg, Russia, Herbert Kroemer at the University of California at Santa Barbara, and Jack Kilby from Texas Instruments in Dallas. Alferov and Kroemer were honored for their work fashioning faster semiconductors that now power international mobile phone networks. Kilby was the first to etch an integrated circuit on silicon wafers — a method that became so common it is now a bit hard to believe someone had to invent it.

The Chemistry Prize went to physicist Alan Heeger of the University of California at Santa Barbara, chemist Alan MacDiarmid of the University of Pennsylvania in Philadelphia, and chemist Hideki Shirakawa of the University of Tsukuba. The three men created electrically conductive plastics that may someday help fuse two usually distinct technologies: television and wallpaper.

*Science* has chosen to lead with a public-private consortium's decision to devote $58 million to sequence the genome the "black six" strain of lab mouse. Led by biotech heavyweights (named by *Nature*; *Science* for some reason didn't name names) SmithKline-Beecham and Merck, the consortium will give away the results free of charge. Most scientists hailed the announcement. Craig Venter, whose company Celera Genomics is in the process of sequencing three other mouse strains, did not. Nature quotes Venter as saying the effort duplicates his company's work and is a waste of public funds. His remedy: spend that cash on licenses to Celera's mouse genome sequence. You know, the one owned by Craig Venter.

Both *Science* and *Nature* devote a full article to comparing the science policies of U.S. Presidential candidates Al Gore and George Bush. In a debate hosted by the American Association for the Advancement of Science, the candidates wisely agreed on one thing: more money for science. But they differ sharply on specifics. Democratic candidate Gore will increase spending in overall basic science focusing most on biomedical (increasing $18 billion over the next 10 years), doubling information technology research over the next 5 years and expanding research in areas that can improve the environment. Republican candidate Bush will also boost biomedical research funding, as well as committing a $20 billion chunk to 5 years of "military research" including eventual deployment of a missile defense. Environmentally speaking, Bush wants to make greenhouse gas reduction voluntary and he will push to get U.S. frankenfoods sold overseas. Neither *Science* nor *Nature* mentioned Ralph Nader.

Which leads naturally to the Ig-Nobels, awarded by the Annals of Improbable Research and covered by *Nature*. Among several awards, American researchers David Dunning and Justin Kreuger took the most election-year-relevant prize for their 1999 paper in psychology, "Unskilled and unaware of it: how difficulties in recognizing one's own incompetence lead to inflated self-assessments."
